# Identification of Alterations in the Expression of Genes Related to the Implant Failure in Spanish Patients with Down Syndrome and Periodontal Disease

**DOI:** 10.3390/genes16020122

**Published:** 2025-01-22

**Authors:** Daniela Cortés-Eslava, Raquel Gómez-Díaz, Daniel Torres-Lagares, Guillermo Machuca-Portillo, José-Luis Gutiérrez-Pérez, María-Ángeles Serrera-Figallo, María Baus-Domínguez

**Affiliations:** 1 Department of Stomatology, Faculty of Dentistry, University of Seville, 41009 Seville, Spain; corteseslavadaniela@gmail.com (D.C.-E.); danieltl@us.es (D.T.-L.); gmachuca@us.es (G.M.-P.); jlgp@us.es (J.-L.G.-P.); maserrera@us.es (M.-Á.S.-F.); 2 Institute of Biomedicine of Seville, 41013 Seville, Spain; rgomez-ibis@us.es; 3 Oral and Maxillofacial Unit, Virgen del Rocio Hospital, 41013 Seville, Spain

**Keywords:** Down syndrome, implant loss, periodontal disease, genetic markers, inflammation, bone metabolism, dental implants

## Abstract

**Background:** Individuals with Down syndrome exhibit a higher prevalence of periodontal disease, which can lead to implant loss. This study aims to identify genetic markers associated with implant loss in these patients, providing insight into potential predictive and therapeutic approaches. **Methods:** A systematic analysis was conducted, including both clinical and genetic data from Down syndrome patients with a history of dental implants. Genetic profiling was performed using Transcriptome Analysis Console (TAC version 4.0 Applied Biosystems^TM^, Thermo Fisher Scientific, Waltham, MA, USA), focusing on genes previously implicated in periodontal disease and bone metabolism. Statistical analysis identified correlations between genetic variants and implant survival rates. **Results:** The analysis revealed statistically significant alterations in several genes related to inflammation and bone remodeling. Key findings included alterations in the expression of the genes *MMP15*, *MMP17*, *S100B*, *GHR*, *DNAH6*, and *ZCCHC14* in patients with implant failure. These genetic markers were strongly correlated with compromised osseointegration and implant loss. These findings underline the role of genetic predisposition in the failure of dental implants among individuals with Down syndrome. **Conclusions:** Genetic markers, particularly those involved in inflammation and bone metabolism, play a critical role in implant loss among Down syndrome patients with periodontal disease. Recognizing these markers can aid in early diagnosis and personalized treatment strategies, potentially improving implant success rates.

## 1. Introduction

Down syndrome (DS) is a genetic condition caused by an error in cell division during meiosis, known as nondisjunction, which usually results in a complete or partial trisomy of chromosome 21. This trisomy is present in 95% of cases, although it can also arise due to mosaicism (2%), involving trisomic and euploid cell lines, or partial trisomy (2–4%) associated with chromosomal rearrangements of chromosome 21 [1,2]. This error occurs mainly during the first meiotic division in the maturing oocyte, with only 10% of cases of paternal origin [2]. According to the UN, the global incidence of DS is between 1 in every 1000 to 1110 newborns [3].

DS is characterized by a complex phenotype with manifestations affecting the neurological, cardiovascular, and musculoskeletal systems [4]. Standard features include short stature, muscular hypotonia, intellectual disability, and congenital heart defects, along with an increased predisposition to diseases such as hypothyroidism, epilepsy, recurrent infections, and hematological disorders. Numerous studies have documented oral alterations in people with DS, such as a reduced midfacial third, narrow palate, delayed dental eruption, absence of teeth, and malformations in dental morphology [4,5]. In addition, the immunologic dysfunction characteristic of this syndrome predisposes to oral infections, which can aggravate systemic conditions [6].

The new classification of periodontal and peri-implant diseases proposed by the American Academy of Periodontology (AAP) and the European Federation of Periodontology (EFP) in 2018 classifies DS as a systemic disease with chronic and progressive manifestations affecting the periodontal junction and alveolar bone [7,8]. In patients with DS, periodontitis usually presents with early onset and increased severity, associated with dysfunctions of the innate and adaptive immune system, such as T-cell lymphopenia, reduced antibody response, and alterations in neutrophil chemotaxis and phagocytosis [9]. In addition, elevated levels of proteolytic enzymes and inflammatory factors contribute to tissue degradation, chronic inflammation, and oxidative stress, increasing susceptibility to periodontal diseases in this group [10]. These complications frequently culminate in early tooth loss, which, combined with intellectual disability, has prompted the use of dental implants as a therapeutic option [11]. Removable prostheses are often discouraged due to the risk of ingestion and the challenges associated with their fabrication and placement [12].

Although the literature indicates that implant failure in patients with DS is related to the unique characteristics of this syndrome, previous studies performed by our working group identified that most failures occur during the period of bone healing before restoration placement. These studies indicated that patients with DS and periodontal disease have a genetic susceptibility to both periodontitis and implant failure [13,14].

Herrera et al. (2023) [15] proposed new guidelines in their systematic review for the prevention and treatment of peri-implant diseases, regardless of patient type. These guidelines can be considered during the perioperative period and the bone healing phase, where patients with Down syndrome have a higher tendency to experience implant loss, aiming to prevent the onset of peri-implantitis. The recommendations range from including the patient in a regular follow-up program to implementing periodontal therapy to eliminate gingival inflammation effectively [16].

Although several authors have investigated implant survival in patients with DS [17,18] and associated marginal bone loss, there is scant literature on the genetic predisposition to this bone loss and its impact on implant survival. This uncertainty highlights the need to investigate the efficacy of dental implant rehabilitation therapies in this population. Therefore, the present study examines a group of patients with DS and periodontal disease to identify significant differences in the expression of genes related to bone metabolism, inflammation, and tissue remodeling, comparing those with and without implant loss, based on the available literature to date. The study considers the conclusions of the 2017 World Workshop of the European Federation of Periodontology/American Academy of Periodontology [19], which established a strong correlation between a history of periodontitis and the subsequent occurrence of peri-implantitis. Furthermore, Serroni et al. (2024) [20] reported in their study a fourfold increased risk of developing peri-implantitis in patients with a prior history of periodontal disease.

Specifically, genes involved in inflammatory response, bone quality, central nervous system development, and growth hormones are evaluated to better understand the genetic basis of implant loss in patients with DS. The analysis aims to explore a potential relationship between these observed genetic alterations and the increased susceptibility to implant loss in DS patients. In the future, considering these findings, therapies could be proposed to enhance the success rates of treatments for this population.

## 2. Materials and Methods

### 2.1. Type of Study

A retrospective case–control study was conducted, approved by the Ethics Committee of the Hospital Universitario Virgen del Rocío (File PI-0081-2016), complying with the ethical principles established in the Declaration of Helsinki of the World Medical Association [21] which regulates medical research on human subjects.

The research, which was descriptive and observational in nature, included only invasive procedures: a dental examination and the collection of a peripheral blood sample for the extraction of genetic material from the participants.

All patients or their legal representatives were informed in detail about the study and gave their informed consent, understanding the possible benefits of the research.

### 2.2. Definition of Groups

This study’s participants were Spanish patients with Down syndrome who attended the dental clinic for their annual check-ups and maintenance of dental implants.

All patients included had a complete and detailed medical history, accompanied by a periodogram and information on the periodontal treatment received and their initial status prior to dental implant placement. In addition, participants had at least two years of follow-up after implant placement and radiographic records, including immediate postoperative radiographs. If a recent radiograph was not available, a new one was taken at the time of sample collection for the study.

The following exclusion criteria, presented in Table 1, were established:

### 2.3. Definition of Variables

#### 2.3.1. Periodontal Disease

The diagnosis of periodontal disease in this investigation was made following the criteria established by Bassani et al. (2007) [21]. For this purpose, six sites per tooth (mesio-vestibular, vestibular, disto-vestibular, mesio-lingual, lingual, and disto-lingual) were evaluated to measure probing depth (PPD) and clinical attachment level (CAL). Periodontal disease was diagnosed in those patients who presented at least three sites in different teeth, with a loss of clinical attachment equal to or greater than 3 mm, accompanied by bleeding on probing (BoP) in the sites examined.

It is important to note that, unlike the study by Bassani et al. (2007) [21], in this study, the severity of periodontitis (mild, moderate, or severe) was not coded, nor were numerical data on probing depth or clinical attachment loss of patients’ teeth included. These data were not considered relevant for the purpose of the study, as the aim was not classifying the type of periodontal disease (Bassani et al. [21]) or determining its grade or stage (Papapanou et al. [6]). Instead, the variable “periodontal disease (PD)” was treated as dichotomous: presence (PD+) or absence (PD−). Similarly, the variable “implant failure” was categorized as present (RI+) or absent (IR−).

Patients were classified as PD− if they had not been diagnosed with periodontal disease in any of their check-ups, including the one performed at the time of sampling, and did not present bleeding on probing in the clinical examination. On the other hand, patients were classified as PD+ if they had been diagnosed with periodontal disease at any of their visits, considering that at the time of implant placement, their periodontitis was treated and inactive or, if they had lost all their teeth, it had been previously diagnosed.

#### 2.3.2. Failure of Dental Implants

To evaluate marginal bone loss (MBL), a study by Lagervall et al. (2013) [22], previously validated for this type of study by Corcuera-Flores et al. (2016) [23], was used. This method is based on the analysis of radiographic records to measure the mesial and distal surfaces of implants not covered by bone. The reference point used is the implant–abutment interface or the most coronal point of the rough surface of the implant. According to this index, implants are classified into four categories:

- Group 0: implants without marginal bone loss.
- Group 1: marginal bone loss of one-third or less of the total length of the implant (bone loss grade 1).
- Group 2: marginal bone loss more significant than one-third, but less than two-thirds of the total length of the implant (bone loss grade 2).
- Group 3: marginal bone loss more significant than two-thirds of the total length of the implant (bone loss grade 3).
- In this study, a fifth group, called Group 4, was added for those patients who had lost the implant.

Patients classified as belonging to the implant failure group (IR+) were those who, after two years of follow-up, presented at least one implant with grade 2 on the Lagervall–Jansson scale.

### 2.4. Laboratory Procedures

#### 2.4.1. Sampling and Isolation of Genetic Material

After the clinical dental examination, patients included in the study had two blood samples obtained using BD Vacutainer™ PAXgene™ tubes (ref. 762,165; Fisher Scientific, Thermo Fisher Scientific Inc., Waltham, MA, USA), which are specifically designed for collecting and storing blood for RNA analysis.

These tubes contain a reagent that ruptures the blood cells and immediately stabilizes the genetic material, preventing its degradation and avoiding alterations in the transcription profile that could arise during storage. This allows the RNA to be preserved without the need for immediate processing.

Collected samples were transported to the Instituto de Biomedicina de Sevilla (IBiS) under refrigeration at 2–8 °C and stored at −20 °C or −80 °C according to requirements. RNA extraction was performed using the PAXgene™ BLOOD miRNA kit (ref. 763,134, QIAGEN, Hilden, Germany), a system designed to stabilize and extract microRNAs (miRNAs), non-coding RNA, and total messenger RNA (mRNA). This process was carried out using QIAGEN’s automated QIAcube station.

Subsequently, the extracted genetic material was quantified using a visible light spectrophotometer, the Thermo Nanodrop 2000C (Ref. ND2000, ThermoScientific, Thermo Fisher Scientific Inc., Waltham, MA, USA), to verify the quality and proper processing of the samples before storage.

Additionally, samples selected for the study were quantified more precisely by fluorescence using the Thermo Qubit 3.0 kit (Ref. Q33216; Invitrogen™ from Life Technologies, Thermo Fisher Scientific, Singapore). This system allows distinguishing between intact and degraded RNA, even in small amounts or in the presence of contaminants, to confirm whether sufficient genetic material is available for analysis.

All data related to the quantification of genetic material were recorded in a database.

#### 2.4.2. Functional Analysis of Expressed Genes

The selected RNA was amplified and hybridized using the Gene-Chip^®^ WT PLUS reagent kit (Thermo Fisher Scientific, Santa Clara, CA, USA), specifically designed for sample preparation in gene expression studies.

The procedure began with the retrotranscription of the extracted mRNA, transforming it into single-stranded cDNA. This cDNA was subsequently hybridized to the array for detailed large-scale gene expression analysis. Amplification was performed from an initial amount of 55 nanograms (ng) of RNA, strictly following the instructions in the GeneChip^®^ WT PLUS kit manuals.

The platform used for this study was the Thermo Scientific GeneChip^®^ Scanner 3000, a high-resolution device designed to analyze single-stranded cDNA microarrays. For this purpose, Clariom S chips specific for humans, mice, and rats were used, covering more than 20,000 genes and allowing accurate measurement of gene expression levels.

Microarray scanning was performed using the GeneChip^®^ Scanner 3000 according to established protocols for array cartridge loading. Finally, data analysis was performed using normalization and the robust multiple arrays (RMA) method. Differential gene expression was analyzed using Transcriptome Analysis Console software (TAC, version 4.0, Applied Biosystems™, Thermo Fisher Scientific, Waltham, MA, USA). This software transforms the intensities of the fluorescent signals detected on the arrays into digital data, which are then used for detailed gene comparisons and analysis.

#### 2.4.3. Statistical Analysis of Reference Genes

Differential gene expression analysis was performed using Transcriptome Analysis Console software (TAC version 4.0, Applied Biosystems™, Thermo Fisher Scientific, Waltham, MA, USA). The genes selected for reference in the present investigation were those identified in relevant articles related to the modulation of the inflammatory response, periodontal disease, and peri-implantitis.

The selection was based on an updated literature search in the PubMed database, using the keywords “implant loss”, “dental implant”, “osseointegration”, “genes or genetics”, “peri-implantitis”, “periodontitis”, and “bone metabolism”. As a result, 11 relevant articles were included [24,25,26,27,28,29,30,31,32,33,34], whose genes are summarized in Table 2.

## 3. Results

### 3.1. Sample Size

Due to strict inclusion and exclusion criteria, only ten patients were selected for the study.

All the patients included were of Spanish nationality and had periodontal disease. Of these, four also had dental implant failure, while the other six showed a favorable evolution of their dental implants after two years of follow-up.

### 3.2. Gene Expression Analysis

Of the 113 genes analyzed in the present gene expression study using Transcriptome Analysis Console software (TAC version 4.0, Applied Biosystems™, Thermo Fisher Scientific, Waltham, MA, USA), only six genes showed statistically significant differences (*p* < 0.05) when comparing patients with Down syndrome who had periodontal disease and implant failure (PD+IR+) with those who had periodontal disease and favorable implant evolution (PD+IR−).

The genes identified were *MMP15*, *MMP17*, *S100B*, *GHR, *DNAH6*,* and *ZCCHC14*. Of these, three showed upregulation, and three showed downregulation (Table 3).

### 3.3. Functional Analysis of Differentially Expressed Genes

The six genes that showed statistically significant differences in expression when comparing the aforementioned groups (PD+RI+ vs. PD+RI−) were analyzed in depth following the methods described in the Retrospective Case–Control Study: Genes Related to Bone Metabolism That Justify the Condition of Periodontal Disease and Failure of Dental Implants in Patients with Down Syndrome [14]. High-impact scientific resources, including the National Center for Biotechnology Information (NCBI) and Online Mendelian Inheritance in Man (OMIM) databases, were used for the analysis, which allowed the identification of functional and associative characteristics of the genes in question.

In addition, the metabolic and molecular pathways involved in these genes were investigated using the Kyoto Encyclopedia of Genes and Genomes (KEGG). Where specific information was unavailable in KEGG, it was supplemented with data extracted from Reactome, a comprehensive database of biological reactions (Table 4). This approach allowed the integration of differential gene expression with their possible roles in key biological processes, such as modulation of bone metabolism, inflammatory responses, and cellular regulation, providing a complete picture of their involvement in periodontal disease and dental implant failure in patients with Down syndrome.

**Figure 1:**
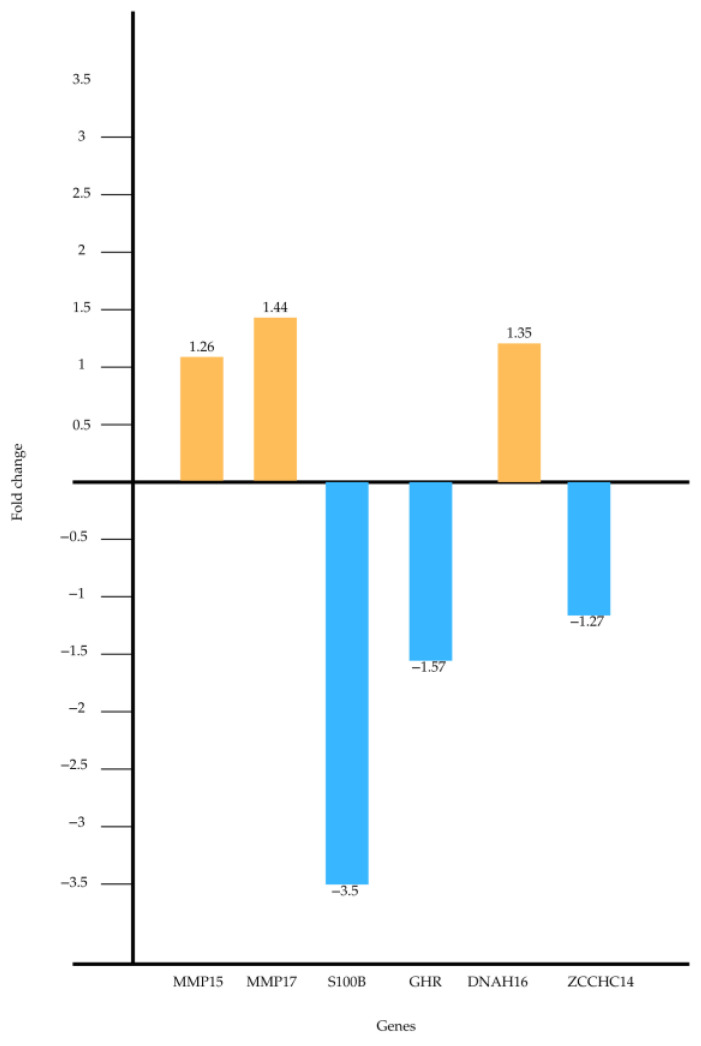
Illustrates the altered fold change in the studied genes. The fold change values represent the relative differences in gene expression between the experimental groups, highlighting genes with statistically significant upregulation or downregulation.

**Figure 2:**
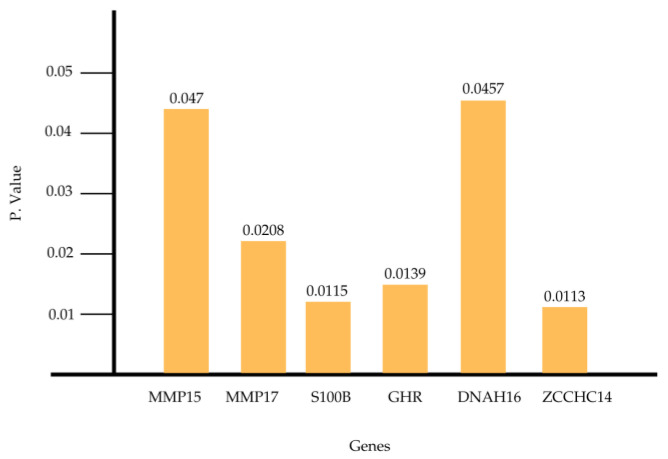
Depicts the *p*-values of the studied genes, indicating the statical significance of the differences in gene expression between the experimental groups. The *p*-values provide a quantitative measure of the likelihood that the observed differences occurred by chance, with lower values representing greater statistical significance (*p* < 0.05).

Figure 1 and Figure 2 provide a visual presentation of the statistically significant altered results.

According to these databases, only *MMP15* and *MMP17* share the synthesis, secretion, and action of parotid hormone; the rest of the genes do not seem to be related in terms of cellular pathways.

## 4. Discussion

Previous research [13] addressed the question of why two groups of individuals with Down syndrome, sharing immunologic and inflammatory alterations characteristic of this syndromic condition, present marked differences in periodontal involvement and dental implant evolution. Specifically, one group presented active periodontal disease and implant failure (PD+RI+), while the other showed no signs of active periodontal disease and achieved favorable implant evolution (PD-RI−). The differential expression of five genes was analyzed: *IL1B*, *IL1RN*, *OCN* (*BGLAP*), *FOXO1*, and *PTK2*, which are related to osseointegration, bone metabolism, healing, and specific functions of osteoblasts and osteoclasts. This finding could, therefore, be related to the bone healing period, where implant failure in patients with Down syndrome is higher. The results indicated that, despite sharing the same baseline genetic condition, patients in the PD+RI+ group present alterations in the expression of key genes involved in bone metabolism through divergent biological pathways. These findings suggest that the PD+RI+ group possesses a particular genetic susceptibility that predisposes it to periodontal disease and dental implant failure [13]. In a complementary study [14], patients with Down syndrome and periodontal disease (DS+PD+) were compared with those without periodontal disease (DS+PD−). Despite having the same syndromic condition, significant differences in susceptibility to periodontal disease were observed. Among the 92 inflammation-related genes evaluated using the TaqMan™ Array Plate Human Inflammation Kit (Thermo Fisher Scientific, Waltham, MA, USA), four genes (*TNFSF13B*, *ITGB2*, *ANXA5*, and *ANXA3*) showed significant differential expression in patients with active periodontal disease.

In a subsequent study [35], patients with PD+RI+ were replaced by those with PD+RI−. Although both groups had active periodontal disease, the genetic results differed. A total of 6 of the 92 genes analyzed showed statistically significant differences (*PLCG2*, *ALOX5*, *LTAH4*, *VCAM1*, *PLA2G2A*, and *PLA2G10*), none of which matched previously identified genes [16]. New genetic alterations emerged upon further comparison between the PD+RI+ and PD+RI− groups. The genes *MMP15*, *MMP17*, *S100B*, *ZCCHC14*, *GHR*, and *DNAH6* were found to be differentially expressed in the PD+RI+ group. This finding highlights the specific genetic influence on the development of periodontal pathologies and dental implant failure, as none of these genes had been reported in previous studies.

The *GHR* and *IGF-1* genes are fundamental regulators of bone homeostasis. They stimulate the proliferation of mesenchymal stem cells (MSCs) and osteoprogenitor cells, promoting their differentiation into osteoblasts and chondrocytes [36,37]. Previous studies have shown that *GHR* inhibits lipogenic genes, favoring osteoblastic differentiation through Wnt signaling, considered a “master switch” in CMS’s commitment to bone formation [38,39]. *GHR* underexpression in the PD+RI+ group could interfere with osteoblastic differentiation, altering bone healing and contributing to implant failure.

For its part, the *S100B* protein regulates cellular processes such as transcription, proliferation, differentiation, and calcium homeostasis, crucial for osteoblasts and osteoclast functions [40]. Its underexpression could reduce bone formation capacity, decreasing bone mineral density, which could explain the implant failures observed in the PD+RI+ group [41]. Although less studied, the *DNAH6* protein is involved in ciliary movement and mesenchymal cell signaling [42]. A recent study showed that *DNAH6* inhibition affects osteoblastic differentiation in Porphyromonas gingivalis-induced periodontitis [43]. Its overexpression in the PD+RI+ group could indicate a compensatory response in bone remodeling.

Likewise, matrix metalloproteinases *MMP15* and *MMP17* are critical enzymes in extracellular matrix degradation. Their imbalanced regulation may contribute to the pathogenesis of periodontitis and periodontal tissue deterioration [44,45,46]. Overexpression of these enzymes in the PD+RI+ group suggests excessive bone remodeling, which, although associated with active inflammation, could prevent bone loss around implants.

Finally, the zinc finger domain protein *ZCCHC14* regulates transcription by binding to RNA or DNA. Studies in osteoarthritis have indicated its involvement in the chondrogenic differentiation of mesenchymal cells [47]. Its underexpression in the PD+RI+ group could explain the observed alterations in bone healing, negatively affecting bone homeostasis and repair.

The study’s limitations include the small sample size inherent to the low prevalence of the condition studied and the strict inclusion criteria. This may limit the generalizability of the findings to larger populations. Furthermore, the implants in the patients included in the sample were not placed as part of the study. Consequently, the risk factor of implant failure due to improper placement, which may contribute to peri-implant disease, was not accounted for. It should also be noted that the control of diseases such as periodontitis and peri-implantitis is polygenic, requiring consideration of the interaction among multiple genes as well as epigenetic factors.

In addition, the study’s cross-sectional nature makes it difficult to establish direct causal relationships between gene expression patterns and observed clinical phenomena. Future research should consider using larger samples and longitudinal designs to validate these results. Exploring the specific roles of the identified genes in the molecular pathways involved in the pathogenesis of periodontal disease and dental implant failure in this population through functional assays could prove valuable. Furthermore, addressing the strong relationship between periodontitis and peri-implantitis, which was not considered in this study, would offer additional insights. It would also be valuable to explore through functional assays the specific role of the identified genes in the molecular pathways involved in the pathogenesis of periodontal disease and dental implant failure in this population. Finally, integrating emerging technologies, such as next-generation RNA sequencing, could provide a more complete picture of gene regulation in these clinical settings.

## 5. Conclusions

The altered genes in the PD+RI+ study group, which present periodontal disease and dental implant failure, share a common participation in metabolic pathways involved in cell differentiation, suggesting an impact on bone healing. This alteration could contribute to implant loss, although it cannot be claimed to be the leading cause of implant failure since the periodontal disease variable present in both the PD+RI+ group and the PD+RI− group has not been considered in depth. Therefore, the findings of this study indicate that the detection of alterations in the aforementioned genes could offer a useful tool for predicting the prognosis of dental implants in patients with Down syndrome, particularly as related to bone healing, allowing progress in the genetic evaluation of the risk of implant failure. However, further studies are required to elucidate whether these genetic alterations directly cause failures or interact with other clinical and systemic factors.

## Figures and Tables

**Table 1:** Exclusion criteria.

Exclusion Criteria
Patients without Down syndrome Patients with Down syndrome but without periodontal disease Patients undergoing treatments that could affect bone metabolism, such as long-term corticosteroids, bisphosphonates, or monoclonal antibodies Patients treated with implants less than 8 mm in size Patients rehabilitated by immediate implant loading Patients with active or untreated periodontal disease at the time of implant placement Patients with no information available on the timing of implant placement or clinical follow-up for at least two years.

**Table 2:** Candidate genes result of the literature search.

Inflammation	Periodontal Disease	Peri-Implantitis
*APOE*, *CCR5*, *CCL4*, *CCL3*, *AKR1D1*, *IFNAR1*, *IFNAR2*, *IL-10*, *STAT1*, *STAT2*, *JAK1*, *MMP8*, *MMP2*, *MMP3*, *MMP9*, *TIMP-2*, *CD14*, *S100A7*, *S100A8*, *S100A9*, *PTGS2*, *IL-6*, *CXCL12*, *FAM3B*, *ITGAL*, *ITGAM*, *ADIPOQ*, *PECAM1*, *FN1*	*APOE*, *CCR5*, *CL4*, *CL3*, *C12orf74*, *KBTBD12*, *PIWIL1*, *C16orf82*, *LHCGR*, *TPR*, *BCR*, *DERL2*, *PLCXD3*, *AKR1D1*, *CDHR4*, *LSM8*, *CCDC60*, *CDCA2*, *GNA12*, *COA4*, *MCHR1*, *BBS12*, *SOD1*, *IFNG*, *IFNAR1*, *IFNAR1*, *IFNGR1*, *IFNGR2*, *IL-10*, *IL-1B*, *STAT1*, *STAT2*, *MMP3*, *MMP9*, *MMP13*, *TIMP-1*, *TIMP-2*, *TIMP3*, *CD4*, *CD8*, *PTGS2*, *HAS2*, *ITGAL*, *CD53*, *PLEK*, *ITGAM*, *SERPIN1*, *APPL1*, *KCNA3*, *LEP*, *ADIPOQ*, *RETN*, *PECAM1*, *CEBPD*, *ADIPOR1*, *NR2F2*, *FN1*, *MPPED1*, *NDEL1*	*CCR5*, *CCL3*, *IL-10*, *IL-1B*, *MMP9*, *CD4*, *PTGS2*, *CD53*, *ADIPOR1*, *FN1*

**Table 3:** Results of the differential gene expression analysis in the two study groups: Down syndrome patients with periodontal disease and implant failure (PD+IR+) and Down syndrome patients with periodontal disease and positive implant evolution (PD+IR−), both after two years of evolution.

Gen	ID Omit	Gene Name	Related to	EP+RI+ AVG (log2)	EP+RI− AVG (log2)	EP+RI+ Standard Deviation	EP+RI− Standard Deviation	Fold Change	*p* Value	Loc. Cytogenetics
*MMP15*	* 602261	Matrix metallopeptidase 15	Bone healing	4.18	3.84	0.13	0.25	1.26	0.0474	16q21
*MMP17*	* 602285	Matrix metallopeptidase 17	Bone healing	6.19	5.68	0.38	0.29	1.44	0.0208	12q24.33
*S100B*	* 176990	S100 calcium binding protein B	Osteoblasts	4.6	6.25	0.07	1.87	−3.5	0.0115	21q22.3
*GHR*	* 600946	Growth hormone receptor	Osseointegration Loss of implants	3.98	4.63	0.38	0.43	−1.57	0.0139	5p13.1-p12
*DNAH6*	* 603336	Dynein, axonemal, Heavy chain6	Osteoclasts	4.75	4.32	0.22	0.35	1.35	0.0457	2p11.2
*ZCCHC14*	* 620697	Zinc finger ccc domain— Containing protein 14	Cell differentiation	7.24	7.58	0.22	0.18	−1.27	0.0113	16q24.2

* The asterisk before the number is the identification format presented by OMIM.

**Table 4:** Summary of the cellular pathways in humans for each gene with a statistically significantly altered result.

Gen	Cellular Pathways in Humans (kegg)	Fold Change	*p* Value
*MMP15*	Parotid hormone synthesis, secretion, and action	1.26	0.0474
*MMP17*	Parotid hormone synthesis, secretion, and action	1.44	0.0208
*S100B*	No data available on KEGG	−3.5	0.0115
*GHR*	(1) Interaction between cytokines and cytokine receptors (2) Interaction between neuroactive ligand and receptor (3) PI3K-Akt signaling pathway (4) JAK-STAT signaling pathway (5) Synthesis, secretion, and action of growth hormone	−1.57	0.0139
*DNAH6*	(1) Motor proteins (2) Amyotrophic lateral sclerosis (3) Huntington’s disease (4) Neurodegeneration pathways—multiple diseases	1.35	0.0457
*ZCCHC14*	No data available on KEGG	−1.27	0.0113

## Data Availability

The original contributions presented in this study are included in the article. Further inquiries can be directed to the corresponding author.
